# Correction: Tuberculosis in advanced chronic kidney disease: An Observational Study at a Tertiary Care Center in Mexico

**DOI:** 10.1371/journal.pone.0356043

**Published:** 2026-08-12

**Authors:** The PLOS ONE Staff

## Notice of Republication

This article was republished on June 17, 2026, to correct errors in the names of the sixth and seventh authors. The publisher apologises for these errors. The correct names are Bernardo Alfonso Martínez-Guerra and Maria Fernanda González-Lara. The correct citation is: Hernández-Ibarra JA, Mercado-Torres TR, Ruiz-Ruiz JR, Román-Montes CM, Rajme-López S, Martínez-Guerra BA, et al. (2026) Tuberculosis in advanced chronic kidney disease: An Observational Study at a Tertiary Care Center in Mexico. PLoS One 21(3): e0338570. https://doi.org/10.1371/journal.pone.0338570.

## Supporting information

S1 File Originally published, uncorrected article. (PDF)

S2 File Republished, corrected article. (PDF)
