# Supplementary material for: Tuberculosis in advanced chronic kidney disease: An Observational Study at a Tertiary Care Center in Mexico
Source: PLoS One. 2026 Mar 20;21(3):e0338570. doi: 10.1371/journal.pone.0338570 (PMC13004396; doi:10.1371/journal.pone.0338570)
Supplement: S1 Table — (DOCX) [file pone.0338570.s001.docx]

Supplementary Table 1.

**Characteristics and Outcomes of Tuberculosis in HIV-Negative Patients, Stratified by Advanced Chronic Kidney Disease Status**

| **Variable** | **Total HIV-Negative Cohort (N=44)** | **ACKD + TB (n=17)** | **TB without ACKD (n=27)** | **p-value** |
| --- | --- | --- | --- | --- |
| **Demographics** |  |  |  |  |
| Median age, years (IQR) | 42 (26-61) | 44(31-61) | 42 (25-60) | 0.596 |
|  |  |  |  |  |
|  |  |  |  |  |
| **Comorbidities** |  |  |  |  |
| Diabetes mellitus, n (%) | 12 (27.3) | 6 (35.3) | 6 (22.2) | 0.343 |
| Heart failure, n (%) | 6 (13.7) | 3 (17.6) | 3 (11.1) | 0.538 |
| Connective tissue disease, n (%) | 14 (31.8) | 5 (29.4) | 9 (33.3) | 0.786 |
| **TB Characteristics** |  |  |  |  |
| Disseminated TB, n (%) | 27 (61.4) | 8 (47.1) | 19 (70.4) | 0.122 |
| Pulmonary TB only, n (%) | 10 (22.7) | 6 (35.3) | 4(14.8) | 0.114 |
| Extrapulmonary TB only, n (%) | 7 (15.9) | 3 (17.7) | 4 (14.8) | 0.803 |
| Positive culture, n/N (%) | 41 (93.2) | 15 (88.2) | 26 (96.3) | 0.302 |
| Positive smear microscopy, n (%) | 11 (25) | 3 (17.7) | 8 (29.6) | 0.371 |
| *M. bovis* infection, n/N (%) | 20/41 (48.8) | 6/15 (40.0) | 14/26 (53.9) | 0.393 |
| *M. tuberculosis* infection, n/N (%) | 21/41 (51.2) | 9/15 (60.0) | 12/26 (46.2) |  |
| Any drug resistance, n/N (%) | 5/41 (12.2) | 2/15 (13.3) | 3/26 (11.6) | 0.866 |
| **Treatment Regimens** |  |  |  |  |
| Conventional daily regimen, n/N (%) | 19 (43.2) | 2 (11.8) | 17 (63) | 0.001 |
| Alternating HD regimen, n/N (%) | 12 (27.3) | 12 (70.6) | 0 (0.0) | <0.001 |
| RHZE + Moxifloxacin, n (%) | 5 (11.4) | 1 (5.9) | 4 (14.8) | 0.363 |
| **Adverse Effects** |  |  |  |  |
| Hepatotoxicity, n (%) | 3 (6.8) | 1 (5.9) | 2 (7.4) | 0.845 |
| Other adverse effects, n (%) | 8 (18.2) | 2 (11.8) | 6 (22.2) | 0.381 |
| **Outcomes** |  |  |  |  |
| Cure, n (%) | 38 (86.4) | 15 (88.2) | 23 (85.2) | 0.774 |
| Relapse, n (%) | 0 (0.0) |  |  |  |
| One-year all-cause mortality, n (%) | 8 (18.2) | 3 (17.7) | 5 (18.5) | 0.942 |
| TB-attributable mortality, n (%) | 3 (7) | 0 (0.0) | 3 (11.1) | 0.167 |

**Notes:**

1. Denominators may vary due to missing data for specific variables (indicated as n/N).
2. p-values calculated using Fisher's exact test for categorical variables and Mann-Whitney U test for continuous variables.
3. ACKD = advanced chronic kidney disease (eGFR <30 mL/min/1.73 m²)
4. RHZE = rifampin, isoniazid, pyrazinamide, ethambutol
