# Supplementary material for: Tuberculosis in advanced chronic kidney disease: An Observational Study at a Tertiary Care Center in Mexico
Source: PLoS One. 2026 Mar 20;21(3):e0338570. doi: 10.1371/journal.pone.0338570 (PMC13004396; doi:10.1371/journal.pone.0338570)
Supplement: S2 Table — (DOCX) [file pone.0338570.s002.docx]

Supplementary Table 2. **Bivariate analysis of factors associated with all-cause mortality.**

| **Variable** | **Survivors (n=42)** | **Deceased (n=9)** | **OR (95%CI)** | **p-value** |
| --- | --- | --- | --- | --- |
| ACKD - n (%) | 14 (33.3) | 3 (33.3) | 1.0 (0.22-4.61) | >0.99 |
| Age, years - Median (IQR) | 42 (30-61) | 29 (22-55) | 0.98 (0.94-1.02) | 0.387 |
|  |  |  |  |  |
| Type 2 diabetes | 10 (23.8) | 2 (22.2) | 0.91 (0.16-5.13) | 0.919 |
| Heart failure | 5 (11.9) | 2 (22.2) | 2.11 (0.34-13.15) | 0.422 |
| Rheumatologic disease - n (%) | 9 (21.4) | 5 (55.6) | 4.58 (1.01-20.69) | 0.048 |
| Any-cause immunosuppression | 7 (77.8) | 10 (23.8) | 11.2 (1.99-62.82) | 0.006 |
| HIV infection | 6 (14.3) | 1 (11.1) | 0.75 (0.08-7.13) | 0.802 |
| Hepatotoxicity - n (%) | 1 (2.4) | 3 (33.3) | 20.5 (1.82-230.52) | 0.014 |
| Other adverse events | 8 (19.1) | 0 | - | 0.154 |
| Disseminated TB - n (%) | 25 (59.5) | 8 (88.9) | 5.44 (0.62-47.56) | 0.126 |
| Conventional regimen | 21 (50) | 4 (44.4) | 0.80 (0.19-3.40) | 0.763 |
| Alternating regimen | 11 (26.2) | 1 (11.1) | 0.35 (0.04-3.15) | 0.350 |
| Moxifloxacin addition | 4 (9.5) | 2 (22.2) | 2.71 (0.41-17.78) | 0.298 |
| Drug resistance | 4 (10.5) | 2 (22.2) | 2.43 (0.37-15.95) | 0.356 |
| Positive smear | 9 (21.4) | 2 (22.2) | 1.05 (0.18-5.94) | 0.958 |

IQR, interquartile range; ACKD, advanced chronic kidney disease; TB, tuberculosis. P-values were calculated using the Mann-Whitney U test for age and Fisher's exact test for categorical variables.
