# Supplementary material for: Correction: Tuberculosis in advanced chronic kidney disease: An Observational Study at a Tertiary Care Center in Mexico
Source: PLoS One. 2026 Aug 12;21(8):e0356043. doi: 10.1371/journal.pone.0356043 (PMC13466020; doi:10.1371/journal.pone.0356043)
Supplement: Supplementary file 2 [file pone.0356043.s002.pdf]

PLOS One

RESEARCH ARTICLE

Tuberculosis in advanced chronic kidney disease: An Observational Study at a Tertiary Care Center in Mexico

José Arturo Hernández-Ibarra[1¶], Tomás Rafael Mercado-Torres[2], José Raúl Ruiz-Ruiz[2], Carla M. Román-Montes[2&], Sandra Rajme-López[2&], Bernardo Alfonso Martínez-Guerra[2&], Maria Fernanda González-Lara[2¶], Héctor O. Rivera-Villegas[2&], Zohar Guy[2], Adriana del C. Roblero-Abadía[2], Alfredo Ponce de-León[2], Jose Sifuentes-Osornio[3], Karla M. Tamez-Torres[2]*

1 Internal Medicine Department, Instituto Nacional de Ciencias Médicas y Nutrición, "Salvador Zubirán", Mexico City, Mexico, **2** Infectious Diseases Department, Instituto Nacional de Ciencias Médicas y Nutrición, "Salvador Zubirán", Mexico City, Mexico, **3** General Direction, Instituto Nacional de Ciencias Médicas y Nutrición "Salvador Zubirán", Mexico City, Mexico,

¶ These authors contributed equally to this work.
& CMR-M, SR-L and BAM-G also contributed equally to this work.
* karla.tamezt@incmnsz.mx

OPEN ACCESS

Citation: Hernández-Ibarra JA, Mercado-Torres TR, Ruiz-Ruiz JR, Román-Montes CM, Rajme-López S, Martínez-Guerra BA, et al. (2026) Tuberculosis in advanced chronic kidney disease: An Observational Study at a Tertiary Care Center in Mexico. PLoS One 21(3): e0338570. https://doi.org/10.1371/journal.pone.0338570

Editor: Ramya Iyadurai, Christian Medical College Vellore, INDIA

Received: November 24, 2025

Accepted: February 15, 2026

Published: March 20, 2026

Copyright: © 2026 Hernández-Ibarra et al. This is an open access article distributed under the terms of the Creative Commons Attribution License, which permits unrestricted use, distribution, and reproduction in any medium, provided the original author and source are credited.

Data availability statement: All relevant data are within the paper and its Supporting Information files.

Abstract

Background

The management of Tuberculosis (TB) in patients with chronic kidney disease (CKD) presents unique challenges, including an immunosuppressive state, altered drug pharmacokinetics, and limited access to single-drug formulations in our setting. There is a scarcity of real-world evidence on TB outcomes in this population in Latin America. Our study aimed to compare mortality, cure, and relapse rates between TB patients with ACKD and without ACKD.

Methods

We conducted an observational-analytical study of all patients aged ≥18 years with microbiologically or histologically confirmed TB between 2013 and 2024. Patients with ACKD (GFR < 30 mL/min/1.73 m² were compared against age- and sex-matched non-ACKD (GFR ≥ 30 mL/min/1.73 m²) patients. Due to differential HIV distribution, we also performed a sensitivity analysis excluding HIV-positive patients. The primary outcome was all-cause mortality at 1 year. Outcomes were compared using the Chi-squared and Mann-Whitney U tests, as well as logistic regression.

Results

A total of 51 patients with tuberculosis were included (17 with ACKD, 34 without ACKD). CKD was caused by lupus or diabetes in 29% of patients each. Most CKD patients (68%) received a local pragmatic alternating regimen. One-year all-cause

Funding: The author(s) received no specific funding for this work.

Competing interests: The authors have declared that no competing interests exist.

mortality was 18% in both groups ($p > 0.999$), and TB-related mortality was 9% in the control group, vs 0% in the ACKD group. The cure rate was similar between groups (ACKD: 88% vs. non-ACKD: 82%; $p = 0.586$). No relapses occurred. In a sensitivity analysis excluding HIV-positive patients ($n = 44$), findings were consistent with the primary analysis, with no significant difference in mortality between groups. Due to the low event rate, we conducted a bivariate analysis in an exploratory fashion and did not perform a multivariate analysis.

Conclusions

In this Mexican small cohort, ACKD didn't significantly worsen TB outcomes compared with non-ACKD patients. An local pragmatic alternating regimen was used without apparent harm. These preliminary findings are limited by small sample size, limited statistical power, and lack of pharmacokinetic validation. Larger studies with drug monitoring are needed to optimize treatment for this vulnerable group.

Introduction

Globally, around 10.8 million people developed tuberculosis in 2023 (134 per 100,000), with approximately 1.25 million deaths, emphasizing TB's continued status as a leading cause of infection-related death; the Americas region contributed with approximately 3.2% of incident cases [1,2].

Chronic kidney disease (CKD), including Advanced CKD (ACKD, eGFR < 30 mL/min/1.73 m²) has been associated with worse outcomes, greater risk for needing renal replacement therapy (RRT), polypharmacy, and a higher risk of infection due to immune dysfunction [3]. People with CKD stages 3–5 have an approximately 57% higher adjusted risk of TB than those without CKD, with the most significant excess risk occurring in stages 4–5 [4].

Clinical presentation in CKD patients is often atypical and insidious. Classic systemic symptoms such as anorexia and weight loss can be attributed to those caused by uremia and thus delay the diagnosis of tuberculosis. Additionally, up to 60–80% of these patients present with extrapulmonary disease, including peritoneal tuberculosis, often diagnosed in the context of peritoneal dialysis-related peritonitis [5]. Notably, only 4–25% of extrapulmonary tuberculosis cases achieve microbiologic confirmation, further underscoring the diagnostic challenge.

The management of TB in CKD represents significant challenges. Pyrazinamide and ethambutol are renally cleared and thus require dose modification. Rifampin has extensive drug interactions and poor penetration to peritoneal fluid, and rifabutin is not available in our region. In CKD patients, individualized non-daily administration of pyrazinamide and ethambutol, careful management of drug interactions, and therapeutic drug monitoring where feasible are recommended [6–9]. In our setting, an intensive-phase (rifampicin 150 mg/isoniazid 75 mg/pyrazinamide 400 mg/ethambutol 300 mg per tablet) and a maintenance-phase (rifampicin 300 mg/isoniazid 400 mg per

tablet) fixed-dose formulations are universally used. Single formulations of pyrazinamide and ethambutol are unavailable and scarce, respectively.

While fixed-dose coformulations remain practical, a problem arises when dose adjustments are required based on toxicity, weight, and/or renal or hepatic function.

In our institution, because of the previously addressed limitations, in the last 10 years, it has been frequently used, what we call an "alternating hemodialysis dosing", in which, during the intensive phase, the patient is instructed to take the fixed-dose formulation of 150 mg rifampicin/75 mg isoniazid/400 mg pyrazinamide/300 mg ethambutol, 3 or 4 tablets based on weight on the days without hemodialysis, and a fixed-dose 300 mg rifampicin/400 mg of isoniazid 2 tablets on the days of hemodialysis, after the session (usually three times a week). This would mean that, in the intensive phase, a 70 kg adult suffering from ACKD and receiving HD three times per week, would be taking pyrazinamide 23 mg/kg 3 times per week, 17 mg/kg of ethambutol 3 times per week, rifampicin 600 mg daily, and isoniazid 300 mg 3 times per week, 800 mg 4 times per week.

Evidence from Latin America on clinical outcomes and the feasibility of pragmatic dosing strategies for TB in patients with ACKD remains scarce. Therefore, this study aimed to describe the rates of all-cause mortality, cure, and relapse in patients with TB with or without ACKD.

Materials and methods

We conducted an observational, analytical study at a national tertiary referral center in Mexico City. Using the Clinical Microbiology and Pathology databases, we identified all specimens positive for Mycobacterium tuberculosis complex (MTBC) by culture, GeneXpert® assay, or histopathology suggestive of tuberculosis between January 2013 and December 2024.

All available adult patients (≥18 years) with microbiologically or histologically confirmed TB who had advanced chronic kidney disease (ACKD; eGFR < 30 mL/min/1.73 m² by CKD-EPI 2021) were included. These cases were compared to patients with preserved renal function (eGFR ≥ 30 mL/min/1.73 m²), matched at a 1:2 ratio by age (±5 years) and sex. We excluded patients with incomplete medical records or those who died within two weeks of TB diagnosis.

To address the differential distribution of HIV infection between groups, a post-hoc sensitivity analysis excluding all HIV-positive patients was conducted.

TB confirmation required at least one of the following: MTBC culture (with species identification by Hain GenoType® or MALDI-TOF), Xpert MTB/RIF® or Ultra assay, tissue IS6110 PCR, or compatible histopathology.

Electronic medical records and clinical charts were reviewed by trained study personnel to gather demographic, microbiological, and clinical information. Patients were followed up for 12 months. Data were accessed on February 2nd 2024. The authors had access to information that could identify individual participants during data collection.

A total of 483 TB cases were identified from the Microbiology Laboratory and Pathology Database from 2013 to 2024. We then calculated the eGFR for all subjects in the 2 months before tuberculosis diagnosis. Then, we excluded 181 patients due to insufficient information (n = 177) or acute renal injury (n = 4) and identified 17 cases with eGFR < 30ml/min and 251 without CKD. From this latter group, we randomly selected 34 non-ACKD subjects, matching them by age and sex with the ACKD patients (Fig 1).

Ethical considerations. Institutional Review Board (Research Ethics and Research Comittees; *Comité de Ética en Investigación y Comité de Investigación*) with local reference number 4922. Informed consent was waived due to the study's retrospective nature.

Definitions

Primary outcome was 1-year all-cause mortality; secondary outcomes: TB-attributable mortality, cure, relapse, and adverse events (AEs). Cure and relapse were adjudicated according to standard definitions and were resolved by consensus [8,10].

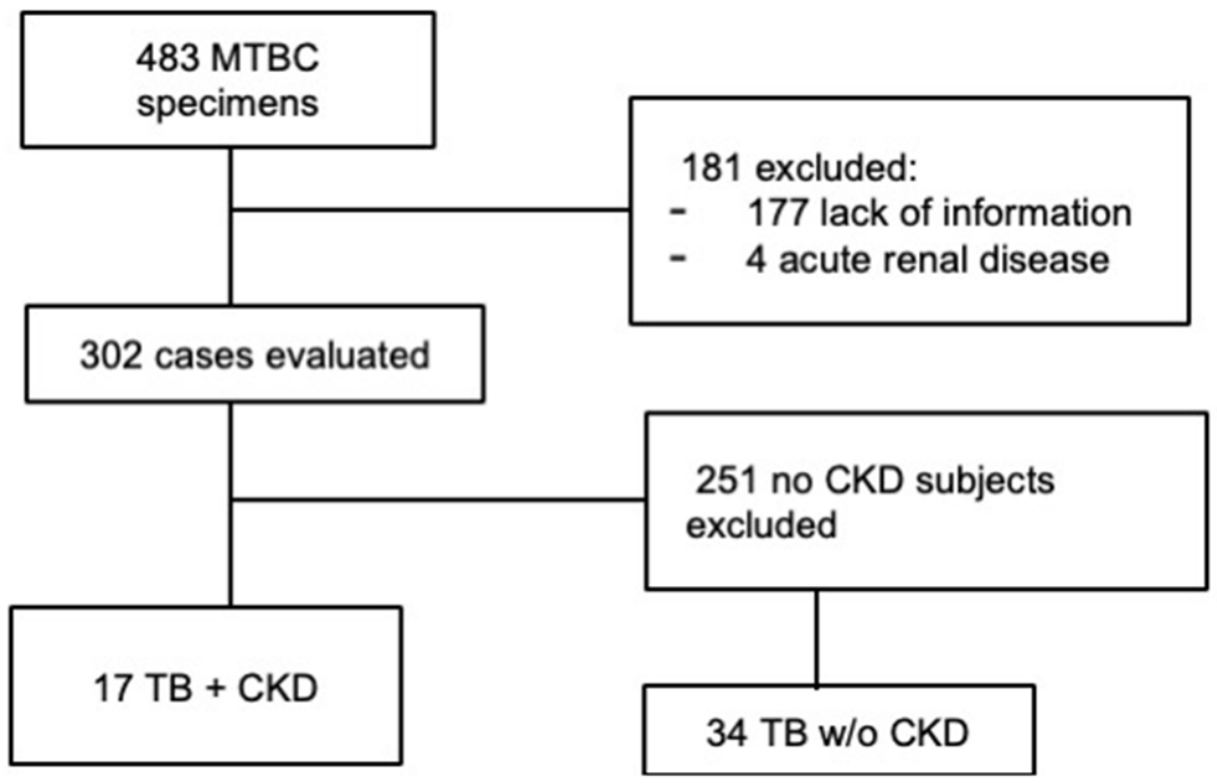

Fig 1. STROBE Diagram of the study.

https://doi.org/10.1371/journal.pone.0338570.g001

Disseminated TB: ≥ 2 non-contiguous sites. Extrapulmonary TB: infection of any organ outside the lungs and pleural space. Hepatotoxicity: ALT ≥ 3 × ULN with symptoms or ≥5 × ULN without symptoms [11]. All patients had serum creatinine and eGFR calculated at the time of tuberculosis diagnosis (or 2 months prior). For each patient, the EMR and creatinine longitudinal data were thoroughly reviewed to eliminate patients with acute renal injury and confirm chronicity.

At our institution, after consensus within the ID Department and due to limited availability of single-drug formulations, we use the previously described local pragmatic alternating regimen.

Statistical analysis

We performed descriptive statistics. Continuous variables are summarized with medians (IQR) and compared using the Mann–Whitney U test, and categorical variables with Fisher's exact test. Due to the small sample size, our study was underpowered to detect small-to-moderate differences in mortality. We performed two sets of analyses: [1] the primary analysis including all patients (n = 51), and [2] a sensitivity analysis excluding HIV-positive patients (n = 44) to address potential confounding by HIV status.

Bivariate analysis was performed exploratorily, and multivariable modeling was not performed due to the limited number of events (n = 9), two-sided α = 0.05.

A priori sample size calculation indicated that 87 ACKD cases and 174 controls would have been required to detect a 15% absolute difference in mortality (30% vs 15%) with 80% power at α = 0.05. The final sample of 17 ACKD cases and 34 controls provided only 26% power to detect a small-to-moderate effect, limiting our ability to detect moderate effects. Analyses were done with Stata v14. (StataCorp LLC, Texas, USA).

Results

We included 51 patients with tuberculosis, 17 with ACKD and 34 without ACKD. Median age 42.4 years (IQR 26–61); 37% were male. Diabetes was documented in 35% (6/17) of ACKD patients compared to 18% (6/34) of non-ACKD patients (p = 0.161). HIV was present only in the control group (21%, 7/34, p = 0.044), and rheumatologic diseases were reported in 29% (5/17) of ACKD versus 24% (8/34) of non-ACKD (p = 0.650). A total of 17 patients met the criteria for ACKD and TB and were compared to 34 TB patients without ACKD. Baseline characteristics and comorbidities are detailed and contrasted in Table 1.

Leading etiologies of ACKD were lupus nephritis with 29% (5/17) and diabetic kidney disease, also with 29% (5/17). Most patients received renal replacement therapy (RRT) (94%, 16/17): hemodialysis (HD) in 82% (14/17) and peritoneal dialysis (PD) in 12% (2/17). 24% (4/17) of patients had a prior kidney transplant with a failed allograft.

Table 1. Demographic characteristics and main comorbidities in patients with tuberculosis, with or without underlying ACKD.

Characteristic	Total (N = 51)	TB +ACKD (n = 17)	TB non-ACKD (n = 34)	p-value
Age, years – Median (IQR)	42.4 (26-61)	43.8 (31-61)	41.8 (25-60)	0.596
Male sex – n (%)	19 (37.3)	7 (41.2)	12 (35.3)	0.682
Female sex	32 (62.7)	10 (58.8)	22 (64.7)	
Type 2 diabetes (T2D) – n (%)	12 (23.5)	6 (35.3)	6 (17.6)	0.161
HIV – n (%)	7 (13.7)	0 (0)	7 (20.6)	0.044
Heart failure (HF) – n (%)	7 (13.7)	3 (17.6)	4 (11.8)	0.565
Rheumatologic disease – n (%)	14 (27.5)	5 (29.4)	9 (26.5)	0.824
Any-cause immunosuppression	17 (33.3)	6 (35.3)	11 (32.3)	0.834

Abbreviations: ACKD, advanced chronic kidney disease; IQR, interquartile range; HIV, Human immunodeficiency virus.

https://doi.org/10.1371/journal.pone.0338570.t001

The lung was the most frequent site of TB in our study; 75% (12/17) of ACKD patients had pulmonary involvement, with the remainder having exclusively extrapulmonary disease. Disseminated TB was observed in 47% (8/17) of ACKD patients and 74% (25/34) of the non-ACKD group.

Outcomes, disease patterns, microbiology, drug regimens, and adverse effects are shown in Table 2. Cure was documented in 88% (15/17) of the ACKD group vs. 82% (28/34) of the non-ACKD group ($p = 0.586$), and relapse occurred in 0% vs 5.9% (2/34) ($p = 0.308$). One-year all-cause mortality was 17.6% (3/17, 6/34) in both groups ($p > 0.99$), and TB-attributable mortality was 0% vs 9% (3/34) ($p = 0.20$).

Disseminated TB occurred in 47% (ACKD) vs 74% (non-ACKD) ($p = 0.062$). Initial culture positivity was 88% in both groups (47/51); smear positivity was 12% (2/17) vs 27% (9/34) ($p = 0.229$). Among the 47 cases with species identification, *M. bovis* accounted for 40% (6/15) in the ACKD-group vs 47% (15/32) in the control group ($p = 0.659$), and *M. tuberculosis* accounted for 60% (9/15) vs 53% (17/32) ($p = 0.659$). Resistance to any antitubercular drug (excluding pyrazinamide resistance in *M. bovis* strains) was observed in 13% of both groups (2/15, 4/32). Among ACKD patients, the local pragmatic alternating regimen was indicated in 71% (12/17). None of the non-ACKD had this regimen indicated. Of the remaining ACKD patients (5/17), two were treated with conventional therapy without dose adjustments, and three (two of whom were on PD) were treated with 3 daily tablets of the intensive-phase multidrug formulation (instead of 4). The other two patients in PD were switched to HD and received the alternating regimen.

Hepatotoxicity occurred in 5.9% (1/17) vs 8.8% (3/34) ($p = 0.713$); the case in the first group led to a temporary suspension of treatment. Other adverse effects, primarily gastrointestinal, were reported in 12% (2/17) of patients vs 21% (7/34) ($p = 0.699$). Grading of events was not performed. Resistance to INH and RIF was observed in two cases, and to streptomycin in the other four.

In the post-hoc sensitivity analysis, the HIV-negative cohort comprised 44 patients: 17 ACKD cases and 27 controls (Supplementary Table 1). In this subset, key outcomes remained similar between groups: one-year all-cause mortality (17.7% vs 18.5%, $p = 0.942$), TB-attributable mortality (0% vs 11.1%, $p = 0.167$), and cure rates (88.2% vs 85.2%, $p = 0.774$). The local pragmatic alternating regimen was used in 70.6% of ACKD patients, compared with 0% of controls ($p < 0.001$), while conventional daily therapy was used in 11.8% of ACKD patients, compared with 63% of controls ($p = 0.001$). (Supplementary Table 2)

The bivariate analysis (Supplementary Table 2) shows that ACKD itself was not associated with mortality (OR 1.0, $p > 0.99$), while rheumatologic disease (OR 4.58, $p = 0.048$) and hepatotoxicity (OR 20.5, $p = 0.014$) were. This reinforces that factors other than renal status drove mortality in your cohort.

Table 2. Outcomes, microbiological data and treatment regimens.

Variable	Total (N = 51)	TB + ACKD (n = 17)	TB non-ACKD (n = 34)	p-value
**Cure – n (%)**	43 (84.3)	15 (88.2)	28 (82.4)	0.586
**Relapse – n (%)**	2 (3.9)	0 (0)	2 (5.9)	0.308
**All-cause mortality – n (%)**	9 (17.6)	3 (17.6)	6 (17.6)	>0.99
**TB-attributable mortality – n (%)**	3 (5.9)	0 (0)	3 (8.8)	0.220
**Disseminated TB – n (%)**	33 (64.7)	8 (47.1)	25 (73.5)	0.062
**Pulmonary TB only, n (%)**	10 (19.6)	6 (35.3)	4 (11.8)	0.046
**Extrapulmonary TB only, n (%)**	8 (15.7)	3 (17.7)	5 (14.7)	0.785
**Positive initial culture – n/N (%)**	47 (92.2)	15 (88.2)	32 (94.1)	0.461
**Positive acid-fast bacilli smear – n/N (%)**	11 (21.6)	3 (17.7)	8 (23.5)	0.630
***Mycobacterium* species – n/N (%)**				
M. bovis	21/47 (44.7)	6/15 (40.0)	15/32 (46.9)	0.659
M. tuberculosis	26/47 (55.3)	9/15 (60.0)	17/32 (53.1)	0.659
**Drug resistance – n/N (%)**	6/47 (12.8)	2/15 (13.3)	4/32 (12.5)	0.936
**Treatment regimen – n/N (%)**				
**Conventional**	25 (49)	2 (12)	23 (67.6)	<0.001
**Hemodialysis-alternating**	12 (23.5)	12 (70.6)	0 (0)	<0.001
**Moxifloxacin addition**	6 (11.8)	1 (5.9)	5 (14.7)	0.357
**Hepatotoxicity – n (%)**	4 (7.8)	1 (5.9)	3 (8.8)	0.713
**Other adverse events – n (%)**	9 (17.6)	2 (11.8)	7 (20.6)	0.699

ACKD, advanced chronic kidney disease

Outcomes were evaluated at 12 months

Disseminated TB: ≥ 2 non-contiguous sites. Extrapulmonary TB: Infection located in any site, except for the lungs and pleura. **Hepatotoxicity**: ALT ≥ 3 × ULN with symptoms or ≥5 × ULN without symptoms.

The **"dialysis-alternating regimen"** refers to the administration of intensive-phase tablet post-hemodialysis and maintenance-phase tablet on non-dialysis days.

Denominators (N) vary due to missing data. P-values were calculated using Fisher's exact test.

https://doi.org/10.1371/journal.pone.0338570.t002

Discussion

Main findings

Among patients treated for TB at a Mexican tertiary center during a 10-year period, ACKD was not linked to increased one-year mortality or lower cure rates compared with matched controls. In this context of limited drug formulation availability, a locally pragmatic, alternating hemodialysis-aligned regimen was used and was not associated with poorer outcomes in our cohort. This experience indicates that structured dialysis-timed administration could be a feasible temporary approach in resource-limited settings where individualized formulations are unavailable, but it requires formal validation. Further studies should assess pharmacokinetics, therapeutic drug monitoring, and comparative effectiveness against guideline-concordant dosing.

Out of 483 TB-positive records from 2013–2023, only 302 (63%) had sufficient clinical data; 181 (37%) were excluded due to incomplete records. The study favors patients with more documentation, often with intensive follow-up. Of these, 51 met all criteria for final analysis. The cohort had a high microbiological confirmation rate (92%), likely due to advanced diagnostics and bias toward accessible samples or severe cases.

TB local epidemiology has been previously studied at our center from 2000 to 2015; 533 cases were evaluated, and 161 (30.2%) were caused by *M. bovis*. Characteristics associated with *M. bovis* disease were younger age, glucocorticoid use, and extrapulmonary disease. [12]

Our center previously evaluated features of extrapulmonary and disseminated tuberculosis in HIV- and non-HIV-related immunosuppression. *M. bovis* caused 44% of 180 TB cases, and more than half of those (54%) didn't have HIV infection [13]. We performed a sensitivity analysis without HIV patients, with similar outcomes, indicating HIV co-infection didn't fully explain our findings; however, we do have a high proportion of immunosuppressed non-HIV patients (around 60% of our patient population has some degree of immunosuppression). We did not eliminate other causes of immunosuppression because the frequency of these factors was balanced between groups. However, the smaller sample size limited statistical power.

Comparison with the literature

Our main finding was that ACKD was not linked to higher one-year mortality than controls (18% vs 18%). This contrasts with studies like Xiao et al. in China, who showed 58% mortality in CKD patients with ACKD on hemodialysis [14], and Vikrant et al. in India, reporting 47% mortality in TB patients on renal therapy [15]. Our control group had high immunosuppression (e.g., 21% HIV), possibly raising baseline risk and reducing outcome differences.

Our findings align with those of Saito et al. in Japan, who found no significant difference in in-hospital TB mortality between CKD and non-CKD patients (3.4% vs 7.1%) [16]. This indicates that timely diagnosis and treatment may reduce the risk of CKD. Our sensitivity analysis excluding HIV-positive patients further suggests ACKD is not an independent predictor of poor outcomes.

Our cohort had a high microbiological confirmation rate (92%) and a high rate of disseminated disease (65%). The 45% of M. bovis reflects advanced diagnostics and immunosuppressed patients, aligning with prior reports [12,13]. In resource-limited settings like Nepal, only 12.3% of CKD patients with TB had microbiological confirmation, often managed empirically due to diagnostic challenges [17].

We found that an alternating hemodialysis regimen may be feasible and not associated with lower cure rates, despite the lack of single-drug formulations of pyrazinamide and ethambutol. Although no direct comparisons are available, our cure rates compare favorably with those of international CKD-TB cohorts. For example, Igari et al. reported a 52.7% cure rate among Japanese hospitalized TB patients with CKD [18]. Our experience shows that structured, dialysis-timed drug administration, despite pharmacokinetic uncertainties, can be a temporary solution in resource-limited settings, ensuring treatment continuity where guideline-based dosing is unfeasible.

No significant differences in adverse events (AEs) were found between the groups. Three patients in the CKD group reported AEs, with only one experiencing hepatotoxicity. Hepatotoxicity was linked to mortality, though causality is unclear.

In a Mexican study, Covarrubias-López et al. reported >80% AE rate in 60 TB patients, mainly renal and hepatic dysfunction; a third of patients were on second-line drugs [19]. In the UK, Quantrill et al. found AEs in 45.8% of CKD-TB patients — hepatotoxicity, gastrointestinal, and neuropsychiatric effects — all in RRT patients [20]. Vikrant et al. noted 29%, mostly GI (12.9%) and hepatotoxicity (12.9%) [16]. In China, Xiao et al. observed higher mild GI and neurotoxic AEs in ACKD patients [14].

There is limited evidence on TB treatment in patients with PD. Hemodialysis regimens may not suit patients with PD; close monitoring of toxicity and serum drug levels is essential. Guidelines suggest using HD doses or switching from PD to HD during TB treatment [8,9].

In our study, we included four patients with PD. One of these patients died during the following year from a cause unrelated to TB, while the rest completed treatment without incidents, and no adverse events were reported in any of them. Further studies are needed in this population to determine the most effective treatment strategies, as our work does not permit drawing solid conclusions.

The primary challenge in treating TB in ACKD in Mexico is the lack of single-drug formulations of pyrazinamide and ethambutol. Our "local pragmatic alternating regimen" was developed to align with hemodialysis schedules and to approximate guideline-recommended, dose-adjusted therapy. This demonstrates feasibility in our setting and shows that treatment in ACKD is achievable even without ideal drug formulations, provided a structured, monitored approach is used.

This study offers real-world evidence on TB drug adjustments in patients with ACKD. It is notable for achieving a high microbiological confirmation rate, a common limitation in extrapulmonary TB, where only 4%−25% are confirmed [14,17]. Additionally, it provides a detailed analysis of ACKD etiologies and includes a control group with other comorbidities, which enhances the validity of the association with mortality. This is the first formal description of an alternative hemodialysis-based TB treatment strategy in Mexico, demonstrating its feasibility and lack of significant safety concerns.

Limitations

Our study has several important limitations. First, its retrospective design and small sample size resulted in limited statistical power (26% to detect the planned 15% mortality difference) and precluded multivariable adjustment for confounders. The wide confidence intervals around our point estimates (e.g., OR 1.0, 95% CI 0.22–4.61) reflect this imprecision, and our finding of 'no difference' should be interpreted as preliminary.

Second, selection bias is likely. A total of 37% of initially identified TB patients were excluded due to incomplete records, and the exceptionally high microbiological confirmation rate (92%), suggests that our cohort represents a selected group of well-characterized cases from a tertiary referral center with advanced diagnostics. This limits the generalizability of our findings to primary care or resource-limited settings, where diagnostic resources and case-mix differ substantially.

Third, regarding the local pragmatic alternating regimen, our study lacked pharmacokinetic data, therapeutic drug monitoring, and a contemporaneous control group receiving fully guideline-concordant therapy [7]. Consequently, we can only report on the feasibility and apparent safety of this regimen in our context, rather than establish comparative efficacy.

Finally, although we attempted to address confounding by HIV through a sensitivity analysis, residual confounding by other forms of immunosuppression (e.g., rheumatologic disease, post-transplant status, chronic steroid use) remains possible. Taken together, these limitations indicate that, in settings where individualized formulations are scarce, a structured, dialysis-timed regimen may be considered a temporary, pragmatic approach until appropriate formulations are available or formal pharmacokinetic and comparative-effectiveness studies are conducted.

Conclusions

In this study, we show that, among patients treated for TB at a Mexican tertiary center, ACKD was not associated with worse 1-year mortality or cure rates than non-ACKD patients. A pragmatic, locally adopted, alternating hemodialysis-aligned regimen was not associated with undesirable outcomes. These findings warrant validation in larger, prospective, multi-center studies.

Supporting information

S1 Table. Characteristics and Outcomes of Tuberculosis in HIV-Negative Patients, Stratified by Advanced Chronic Kidney Disease Status.
(DOCX)

S2 Table. Bivariate analysis of factors associated with all-cause mortality.
(DOCX)

S3 Dataset. Dataset.
(CSV)

S4 Data. Data dictionary.
(TXT)

Author contributions

Conceptualization: Karla M. Tamez-Torres, Jose Arturo Hernández-Ibarra, Maria Fernanda González-Lara, Alfredo Ponce de-León.

Data curation: Karla M. Tamez-Torres, Jose Arturo Hernández-Ibarra, Tomas Rafael Mercado-Torres, Jose Raul Ruiz-Ruiz, Zohar Guy, Adriana del C Roblero-Abadía.

Formal analysis: Karla M. Tamez-Torres, Bernardo Alfonso Martínez-Guerra.

Investigation: Karla M. Tamez-Torres, Jose Arturo Hernández-Ibarra, Tomas Rafael Mercado-Torres, Jose Raul Ruiz-Ruiz, Carla M Román-Montes, Sandra Rajme-López, Bernardo Alfonso Martínez-Guerra, Hector O Rivera-Villegas.

Methodology: Karla M. Tamez-Torres, Jose Arturo Hernández-Ibarra, Maria Fernanda González-Lara, Alfredo Ponce de-León.

Resources: Alfredo Ponce de-León, Jose Sifuentes-Osornio.

Supervision: Maria Fernanda González-Lara, Alfredo Ponce de-León, Jose Sifuentes-Osornio.

Writing – original draft: Karla M. Tamez-Torres, Jose Arturo Hernández-Ibarra.

Writing – review & editing: Karla M. Tamez-Torres, Jose Arturo Hernández-Ibarra, Jose Raul Ruiz-Ruiz, Carla M Román-Montes, Sandra Rajme-López, Bernardo Alfonso Martínez-Guerra, Maria Fernanda González-Lara, Hector O Rivera-Villegas, Zohar Guy, Adriana del C Roblero-Abadía, Jose Sifuentes-Osornio.

References

1. Bennett JE, Dolin R, Blaser MJ. Mandell, Douglas, and Bennett's Principles and Practice of Infectious Diseases E-Book: 2-Volume Set. Elsevier Health Sciences. 2019.
2. Kidney Disease: Improving Global Outcomes (KDIGO) CKD Work Group. KDIGO 2024 Clinical Practice Guideline for the Evaluation and Management of Chronic Kidney Disease. Kidney Int. 2024;105(4S):S117–314. https://doi.org/10.1016/j.kint.2023.10.018 PMID: 38490803
3. Alcazar-Arrollo R. Advanced chronic kidney disease. Nefrol Engl Ed. 2008;28:3–6.
4. Luczynski P, Holmes T, Romanowski K, Arbiv OA, Cook VJ, Clark EG. Risk of tuberculosis disease in people with chronic kidney disease without kidney failure: a systematic review and meta-analysis. Clin Infect Dis Off Publ Infect Dis Soc Am. 2023;77(8):1194–200.
5. Venkata RKC, Kumar S, Krishna RP, Kumar SB, Padmanabhan S, Kumar S. Tuberculosis in chronic kidney disease. Clin Nephrol. 2007;67(4):217–20. https://doi.org/10.5414/cnp67217 PMID: 17474557
6. Saukkonen JJ, Duarte R, Munsiff SS, Winston CA, Mammen MJ, Abubakar I. Updates on the Treatment of Drug-Susceptible and Drug-Resistant Tuberculosis: An Official ATS/CDC/ERS/IDSA Clinical Practice Guideline. Am J Respir Crit Care Med. 2025;211(1):15–33.
7. Alffenaar JWC, Stocker SL, Forsman LD, Garcia-Prats A, Heysell SK, Aarnoutse RE. Clinical standards for the dosing and management of TB drugs. Int J Tuberc Lung Dis. 2022;26(6):483–99.
8. Nahid P, Dorman SE, Alipanah N, Barry PM, Brozek JL, Cattamanchi A, et al. Official American Thoracic Society/Centers for Disease Control and Prevention/Infectious Diseases Society of America Clinical Practice Guidelines: Treatment of Drug-Susceptible Tuberculosis. Clin Infect Dis. 2016;63(7):e147–95. https://doi.org/10.1093/cid/ciw376 PMID: 27516382
9. Ahn C, Oh K-H, Kim K, Lee KY, Lee JG, Oh MD, et al. Effect of peritoneal dialysis on plasma and peritoneal fluid concentrations of isoniazid, pyrazinamide, and rifampin. Perit Dial Int. 2003;23(4):362–7. https://doi.org/10.1177/089686080302300409 PMID: 12968844
10. Organization WH. Framework for collaborative action on tuberculosis and comorbidities. World Health Organization; 2022.
11. Saukkonen JJ, Cohn DL, Jasmer RM, Schenker S, Jereb JA, Nolan CM, et al. An official ATS statement: hepatotoxicity of antituberculosis therapy. Am J Respir Crit Care Med. 2006;174(8):935–52. https://doi.org/10.1164/rccm.200510-1666ST PMID: 17021358
12. Torres-Gonzalez P, Cervera-Hernandez ME, Martinez-Gamboa A, Garcia-Garcia L, Cruz-Hervert LP, Bobadilla-Del Valle M, et al. Human tuberculosis caused by Mycobacterium bovis: a retrospective comparison with Mycobacterium tuberculosis in a Mexican tertiary care centre, 2000-2015. BMC Infect Dis. 2016;16(1):657. https://doi.org/10.1186/s12879-016-2001-5 PMID: 27825312
13. Aceves-Sánchez B, Rajme-López S, Martínez-Guerra BA, Rivera-Villegas H, Román-Montes CM, Tamez-Torres KM. Distinct clinical features of extrapulmonary and disseminated tuberculosis in HIV- and non-HIV-associated immunosuppression: a retrospective cohort study. Open Forum Infect Dis. 2025;12(5):ofaf239. https://doi.org/10.1093/ofid/ofaf239
14. Xiao J, Ge J, Zhang D, Lin X, Wang X, Peng L, et al. Clinical Characteristics and Outcomes in Chronic Kidney Disease Patients with Tuberculosis in China: A Retrospective Cohort Study. Int J Gen Med. 2022;15:6661–9. https://doi.org/10.2147/IJGM.S367090 PMID: 36016982

15. Vikrant S. Tuberculosis in dialysis: Clinical spectrum and outcome from an endemic region. Hemodial Int. 2019;23(1):88–92. https://doi.org/10.1111/hdi.12693 PMID: 30289617
16. Saito N, Yoshii Y, Kaneko Y, Nakashima A, Horikiri T, Saito Z, et al. Impact of renal function-based anti-tuberculosis drug dosage adjustment on efficacy and safety outcomes in pulmonary tuberculosis complicated with chronic kidney disease. BMC Infect Dis. 2019;19(1):374. https://doi.org/10.1186/s12879-019-4010-7 PMID: 31046706
17. Pradhan RR, Sigdel MR. Prevalence, clinical presentation, and outcome of tuberculosis in patients with chronic kidney disease at a tertiary care hospital in Nepal. Int J Nephrol. 2020;2020(1):7401541.
18. Igari H, Imasawa T, Noguchi N, Nagayoshi M, Mizuno S, Ishikawa S, et al. Advanced stage of chronic kidney disease is risk of poor treatment outcome for smear-positive pulmonary tuberculosis. J Infect Chemother. 2015;21(8):559–63. https://doi.org/10.1016/j.jiac.2015.04.008 PMID: 26048063
19. Díaz-Covarrubias T, Laniado-Laborín R. Reacciones adversas a los fármacos antituberculosis en pacientes con esquemas mixtos. Neumología y cirugía de tórax. 2016;2(75):149–54.
20. Quantrill SJ, Woodhead MA, Bell CE, Hardy CC, Hutchison AJ, Gokal R. Side-effects of antituberculosis drug treatment in patients with chronic renal failure. Eur Respir J. 2002;20(2):440–3. https://doi.org/10.1183/09031936.02.00298002 PMID: 12212979
